# H5N1 Influenza Viruses in Lao People’s Democratic Republic

**DOI:** 10.3201/eid1210.060658

**Published:** 2006-10

**Authors:** David A. Boltz, Bounlom Douangngeun, Settha Sinthasak, Phouvong Phommachanh, Scott Rolston, Honglin Chen, Yi Guan, Joseph S. Malik Peiris, Gavin J.D. Smith, Robert G. Webster

**Affiliations:** * St Jude Children's Research Hospital, Memphis, Tennessee, USA;; † National Animal Heath Centre, Vientiane, Lao People's Democratic Republic;; ‡ Embassy of the United States, Vientiane, Lao People's Democratic Republic;; § Shantou University Medical College, Shantou, People's Republic of China;; ¶ University of Hong Kong, Hong Kong Special Administrative Region, People's Republic of China

**Keywords:** Lao PDR, H5N1, Surveillance, Serology, Duck, dispatch

## Abstract

A prospective surveillance program for influenza viruses was established in Lao People's Democratic Republic (PDR) in July of 2005. We report isolation of H5N1 virus genetically distinct from H5N1 circulating in 2004, which indicates reintroduction of H5N1 into Lao PDR after its disappearance (i.e., no virologic or serologic evidence) for 2 years.

H5N1 influenza viruses have continuously circulated in southeastern Asia since 1996 (*1**–**5*). During late 2003 and early 2004, highly pathogenic H5N1 spread throughout China, Vietnam, Thailand, Cambodia, and Lao People's Democratic Republic (PDR) with a high lethality for domestic poultry; whereas in Vietnam, Thailand, and Cambodia, humans were infected but at a lower rate.

In Lao PDR, outbreaks were reported in the provinces of Vientiane Capital, Champasak, and Savannakhét. Approximately 155,000 poultry either died of disease or were culled, which contained the 2003–2004 outbreak. No human cases have been reported, and no scientific evidence has indicated avian influenza cases in poultry in Lao PDR since March 2004. This finding raises some questions. Had H5N1 viruses been eradicated from Lao PDR, or had they survived in domestic waterfowl to which they were nonpathogenic, as Chen et al. described in southern China (*6**,**7*).

In response to outbreaks of H5N1, prospective surveillance programs have been established in affected southeastern Asian countries. In southern China, systematic surveillance of poultry markets has been ongoing since July 2000 (*7*). Thailand launched a comprehensive nationwide surveillance strategy (the x-ray survey) in October 2004 (*8*). Cambodia and Vietnam have also initiated surveillance programs, but little is known about the extent of the programs and their results. In Lao PDR since the outbreak in early 2004, surveillance programs have been limited to confirming the presence or absence of avian influenza. Given the frequency of H5N1 outbreaks in the region, a baseline for surveillance and determination of risk areas was essential. Therefore, in July 2005 we helped the Lao National Animal Health Centre establish a prospective surveillance program. We report the results of an 8-month surveillance program and the apparent reintroduction of H5N1 viruses into Lao PDR in early 2006.

## The Study

**Figure 1:**
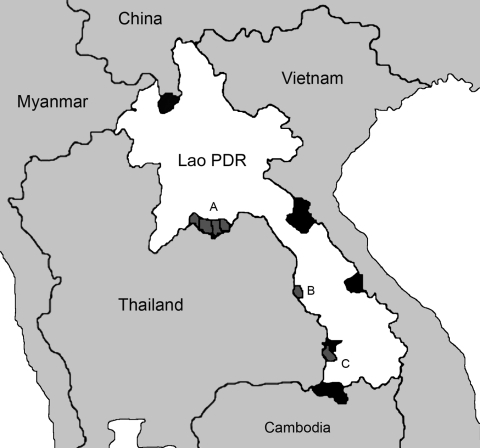
Map of Lao People's Democratic Republic indicates regions (black areas) of influenza virus surveillance. Outbreaks of H5N1 occurred in Vientiane (location of isolation of A/Duck/Laos/3295/2006) (A), Savannakhét (B), and Champasak (C) provinces during late 2003 and early 2004.

Extensive virologic surveillance began in Lao PDR in July 2005. During 8 months, 8,592 samples were collected from healthy ducks, chickens, quail, and pigs at live markets in Vientiane, Champasak, and Savannakhét provinces, near the borders of Thailand, Vietnam, and Cambodia (Figure 1). Cloacal swabs were stored in 1.0 mL of virus isolation media. The samples were injected into chicken or duck eggs, and allantoic fluid was harvested after 72 h and analyzed as described (*9*). As of January 2006, no evidence of any influenza virus subtypes had been detected in any species.

In January 2006, serologic surveillance was initiated to clarify whether poultry in Lao PDR had been exposed to influenza viruses and recovered. Vaccination of poultry is not practiced in Lao PDR, which made serologic surveillance possible. Serologic analysis for antibodies to influenza viruses was performed with ELISA and hemagglutination inhibition (HI). The IDEXX FlockChek AIV (IDEXX Laboratories, Inc., Westbrook, ME, USA) was used to initially screen for antibodies to influenza. Sera were then tested by HI with antigens to H5, H6, H7, and H9 influenza subtypes as described (*10*). Initial results from serologic surveillance confirmed virus isolation results and absence of vaccine use; however, in early February 2006, in apparently healthy ducks in a live market in Vientiane, we detected antibodies to the viral hemagglutinins H5 (HI titers 40 to <640) and H9 (HI titer 80). Only 21 (3.5%) of 604 birds had positive test results at that time; 1 duck had antibodies to H5 and H9.

**Figure 2:**
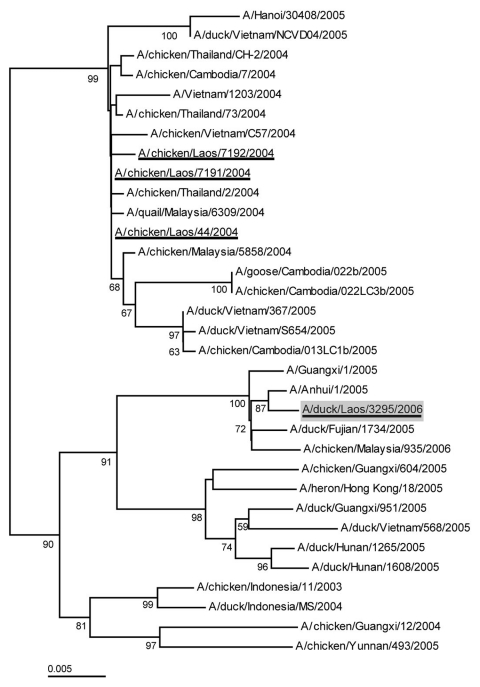
Phylogenetic relationship of the hemagglutinin (HA) gene of representative H5N1 influenza virus strains isolated in Southeast Asia from 2003 through 2006. Analysis was based on nucleotides 1–1012 (1,012 bp) of the HA gene. Viruses isolated in Lao People's Democratic Republic in 2004 and 2006 are underlined. The strain isolated from ducks in Vientiane in February 2006 is shaded.

In late February 2006, H5N1 was isolated from healthy ducks at a layer farm in Vientiane Capital. The source of the isolated H5N1 is unknown, but our 8-month prospective surveillance program showed that it was recently introduced into this infected flock. Cloacal samples from 40 ducks at a duck farm in Vientiane tested negative for H5N1 virus in November 2005, but upon retesting in late February 2006, results for the same flock were positive for H5N1. To identify the source of the new isolate, we compared the sequence of its hemagglutinin gene with those of H5N1 viruses isolated since 2003 in China, Thailand, Vietnam, and Cambodia (Figure 2).

Sequence analysis indicated that the new H5N1 isolate (A/duck/Laos/3295/2006) (GenBank accession no. DQ845348) was not closely related to the other viruses circulating in Southeast Asia during 2003 and 2004. It clustered phylogenetically with H5N1 viruses isolated in China in 2005 and was most closely related to a 2005 human isolate from Anhui, China (A/Anhui/1/2005) (*11*). Furthermore, the 2004 Laotian H5N1 virus strains clustered with 2004 isolates from Malaysia, Thailand, and Vietnam. This evidence suggests that the H5N1 virus had recently been introduced into Lao PDR; however, its direct source is unknown. Despite its origin in China, it clustered with a 2005 Vietnamese virus isolate (A/duck/Vietnam/568/2005) and therefore may have crossed into Lao PDR from Vietnam. A Malaysian H5N1 isolate (A/chicken/Malaysia/935/2006) also clustered with this group rather than with Malaysian isolates from 2004, which indicates that it was likely a new introduction into Malaysia.

Surveillance for H5N1 continued in the area surrounding the infected farm. Two months after the isolation of H5N1, 40 cloacal swabs and 28 serum samples were collected from ducks at the infected and surrounding farms. All cloacal swabs had negative results for influenza viruses, and serum samples had negative results for antibodies to H5N1. As of June 2006, no reports of infected poultry have been made since the isolation, which indicates that H5N1 did not spread and its introduction may have been an isolated event; however, surveillance is ongoing.

## Conclusions

As highly pathogenic H5N1 influenza viruses continue to spread into Eurasia, whether they will become endemic in Eurasian poultry or whether the highly pathogenic H5N1 will burn out and disappear with the possibility of reintroduction is unknown. We describe results from the first active surveillance study in Lao PDR since the 2004 outbreak. We extensively sampled live markets and local farms in Vientiane Capital and additionally sampled districts bordering Cambodia, China, Thailand, and Vietnam. A genetically distinct H5N1 was isolated from apparently healthy ducks, which indicates reintroduction of H5N1 in Lao PDR. Serologic data, antibodies to H5 in serum samples from apparently healthy ducks found in live markets, provide additional evidence of H5N1 in Lao PDR; however, we cannot rule out the illegal use of vaccines. Evidence of an H9 subtype was also detected in a duck that was seropositive for H9 antibodies; however, no H9 influenza subtypes were isolated.

Whether H5N1 influenza viruses are endemic in wild migratory birds is unresolved; however, our study supports the notion that highly pathogenic H5N1 viruses are not endemic in Lao PDR. Our findings suggest that the H5N1 virus did not become established after the 2004 outbreak in Lao PDR, which is less densely populated than neighboring Thailand, Vietnam, and China (high summer temperatures in Lao PDR may have also been a factor). Instead, the virus disappeared and was later replaced by a newly introduced virus. Although no measures were taken to eradicate the virus, the newly introduced H5N1 has since vanished from the area. The area has been identified as high risk and has become the focus of ongoing surveillance.

This virus appears to have disappeared on its own in Lao PDR in the absence of vaccines and with limited surveillance, unlike what happened in the more densely populated Thailand, Vietnam, and China. In China, H5N1 continues to circulate in poultry despite ongoing surveillance and use of vaccines; heroic measures were required to eradicate H5N1 in Thailand, and since the use of vaccines in Vietnam in 2005, no more human cases have been reported.

These findings are encouraging for the less densely populated, developing countries of Africa and Asia because they suggest that H5N1 viruses are not being perpetuated through wild migratory birds. To date, wild birds have not been found to carry and spread highly pathogenic H5 and H7 influenza viruses; instead, each outbreak has been caused by a phylogenetically new virus, whose parent viruses were not pathogenic (*12**,**13*).

## References

[1] Tang X, Tian G, Zhao J, Zhou KY. Isolation and characterization of prevalent strains of avian influenza viruses in China [article in Chinese]. Chinese Journal of Animal and Poultry Infectious Diseases. 1998;20:1–5.

[2] Cauthen AN, Swayne DE, Schultz-Cherry S, Perdue ML, Suarez DL. Continued circulation in China of highly pathogenic avian influenza viruses encoding the hemagglutinin gene associated with the 1997 H5N1 outbreak in poultry and humans. J Virol. 2000;74:6592–9. 10.1128/JVI.74.14.6592-6599.2000 10864673 PMC112169

[3] Guan Y, Peiris JS, Lipatov AS, Ellis TM, Dyrting KC, Krauss S, Emergence of multiple genotypes of H5N1 avian influenza viruses in Hong Kong SAR. Proc Natl Acad Sci U S A. 2002;99:8950–5. 10.1073/pnas.132268999 12077307 PMC124404

[4] Webster RG, Guan Y, Peiris M, Walker D, Krauss S, Zhou NN, Characterization of H5N1 influenza viruses that continue to circulate in geese in southeastern China. J Virol. 2002;76:118–26. 10.1128/JVI.76.1.118-126.2002 11739677 PMC135698

[5] Li KS, Guan Y, Wang J, Smith GJ, Xu KM, Duan L, Genesis of a highly pathogenic and potentially pandemic H5N1 influenza virus in eastern Asia. Nature. 2004;430:209–13. 10.1038/nature02746 15241415

[6] Sturm-Ramirez KM, Hulse-Post DJ, Govorkova EA, Humberd J, Seiler P, Puthavathana P, Are ducks contributing to the endemicity of highly pathogenic H5N1 influenza virus in Asia? J Virol. 2005;79:11269–79. 10.1128/JVI.79.17.11269-11279.2005 16103179 PMC1193583

[7] Chen H, Smith GJ, Li KS, Wang J, Fan XH, Rayner JM, Establishment of multiple sublineages of H5N1 influenza virus in Asia: implications for pandemic control. Proc Natl Acad Sci U S A. 2006;103:2845–50. 10.1073/pnas.0511120103 16473931 PMC1413830

[8] Tiensin T, Chaitaweesub P, Songserm T, Chaisingh A, Hoonsuwan W, Buranathai C, Highly pathogenic avian influenza H5N1, Thailand, 2004. Emerg Infect Dis. 2005;11:1664–72. 16318716 10.3201/eid1111.050608 PMC3367332

[9] Shortridge KF, Butterfield WK, Webster RG, Campbell CH. Isolation and characterization of influenza A viruses from avian species in Hong Kong. Bull World Health Organ. 1977;55:15–9. 302152 PMC2366618

[10] Palmer DF, Dowdle WR, Coleman MT, Schild GC. Advanced laboratory techniques for influenza diagnosis. Immunology series no. 6. Atlanta: Center for Disease Control, US Department of Health, Education and Welfare; 1975.

[11] Shu Y, Yu H, Li D. Lethal avian influenza A (H5N1) infection in a pregnant woman in Anhui Province, China. N Engl J Med. 2006;354:1421–2. 10.1056/NEJMc053524 16571888

[12] Rohm C, Horimoto T, Kawaoka Y, Suss J, Webster RG. Do hemagglutinin genes of highly pathogenic avian influenza viruses constitute unique phylogenetic lineages? Virology. 1995;209:664–70. 10.1006/viro.1995.1301 7778300

[13] Alexander DJ. A review of avian influenza in different bird species. Vet Microbiol. 2000;74:3–13. 10.1016/S0378-1135(00)00160-7 10799774

